# Hydroxychloroquine use is not associated with QTc length in a large cohort of SLE and RA patients

**DOI:** 10.1186/s13075-021-02646-0

**Published:** 2021-10-29

**Authors:** Elizabeth Park, Jon T. Giles, Thania Perez-Recio, Paloma Pina, Christopher Depender, Yevgeniya Gartshteyn, Anca D. Askanase, Joan Bathon, Laura Geraldino-Pardilla

**Affiliations:** 1 Division of Rheumatology, Columbia University Vagelos College of Physicians and Surgeons and New York Presbyterian Hospital, 630 W 168th St, P&S 3-450, New York, NY 10032 USA; 2 Cardiac Electrophysiology, Northwestern Medicine, Chicago, IL USA

**Keywords:** Rheumatoid arthritis, Systemic lupus erythematosus, Hydroxychloroquine, QTc

## Abstract

**Background:**

Hydroxychloroquine (HCQ) is a cornerstone therapy for systemic lupus erythematosus (SLE) and rheumatoid arthritis (RA). However, reports of its use and subsequent fatal arrhythmias in patients with coronavirus disease 19 (COVID-19) have raised concern regarding its cardiovascular (CV) safety. Therefore, we examined the relationship between HCQ use and corrected QT (QTc) length in SLE and RA patients without clinical CV disease (CVD).

**Methods:**

SLE patients from the Columbia University Lupus Cohort registry (*n* = 352) and two RA cohorts (*n* = 178; ESCAPE-RA and RHYTHM-RA) with electrocardiograms (ECGs) collected as part of study data were analyzed. RA cohort participants were recruited from tertiary referral centers with additional referrals from community rheumatologists, while SLE subjects originated from the Columbia University Lupus Cohort. All patients met American College of Rheumatology (ACR) classification criteria for SLE or RA and lacked known CVD. The exposure of interest was HCQ use and main outcome measure was QTc length [continuous or categorical (≥ 440 ms and ≥ 500 ms)].

**Results:**

Of the combined SLE and RA cohorts (*n* = 530), 70% were HCQ users and 44% had a QTc ≥ 440 ms. The adjusted mean QTc length was comparable between HCQ users vs non-users (438 ms vs 437 ms). In multivariable logistic models, HCQ use was *not* a significant predictor of a QTc ≥ 440 ms for the entire cohort (OR 0.77; 95% CI 0.48–1.23; *p* = 0.27). Importantly, a QTc ≥ 500 ms was inversely associated with HCQ use and not associated with arrhythmias or deaths. A significant interaction was found between HCQ use and use of anti-psychotics. Ultimately, the use of HCQ combined with any QTc prolonging medication as a group was associated with a QTc length (434 ms; 95% CI 430, 439) which was comparable to that of use of HCQ alone (433 ms; 95% CI 429-437).

**Conclusion:**

In a combined cohort of SLE and RA patients without clinical CVD, adjusted QTc length was comparable between HCQ and non-HCQ users, supporting its CV safety in patients with rheumatic diseases.

**Supplementary Information:**

The online version contains supplementary material available at 10.1186/s13075-021-02646-0.

## Background

Hydroxychloroquine (HCQ) has long been a cornerstone therapy for systemic lupus erythematosus (SLE) and is commonly used as monotherapy or combined with other disease modifying anti-rheumatic drugs (DMARDs) for rheumatoid arthritis (RA). HCQ neutralizes acidic cytoplasmic components within the lysosome, leading to downstream alterations in antigen processing and inhibition of toll-like receptors [1, 2]. The observed cutaneous, retinal, and musculoskeletal toxicities are thought to arise from long-term storage of its metabolite, 4-aminoquinolone, in these tissues [2].

Acute cardiovascular (CV) toxicities from short-term exposure of HCQ (and subsequent blocking of potassium channels within myocytes) manifest as QT interval prolongation, and when combined with additional baseline risk factors, such as age, sex, high levels of anti-Ro antibodies [3], and arrhythmogenic congenital long QT syndromes [4], acute arrhythmic events (i.e., torsades de pointes) also occur [5]. With long-term exposure, HCQ metabolites may also accumulate in the myocardium and result in a cardiomyopathy with concentric hypertrophy and conduction abnormalities [1, 5, 6]. In fact, 33 of 42 histologically confirmed cases of HCQ induced cardiomyopathy originated from RA and SLE patients, who on average had been on treatment for 13 years [1]. Moreover, 14 of those 42 cases progressed to third-degree atrioventricular block. However, there are no current guideline-based recommendations for CV screening in the setting of prolonged HCQ use.

Recent reports [7, 8] of possible associations between concurrent HCQ and azithromycin use and QTc prolongation in those receiving treatment for coronavirus disease 2019 (COVID-19)-associated pneumonia have raised further concerns for HCQ-associated cardiotoxicity. However, the wide spectrum of cardiotoxic effects of COVID-19 itself (arrhythmias, myocarditis, microvascular injury, stress cardiomyopathy) [9] confound these observations. In observational (mostly retrospective) studies in rheumatic disease patients [10–14], there were no differences in QTc prolongation in HCQ users vs non-users, nor was there an association between QTc length and HCQ use, keeping in mind that these studies did not consistently account for the use of other prolonging QTc medications. We therefore investigated associations between HCQ use and QTc length in an SLE and RA cohort without known CV disease (CVD), accounting for the use of various QTc prolonging medications.

## Methods

### Study population

A total of 530 SLE and RA patients on whom HCQ use information was available were included in the study.

### SLE patients

**Fig. 1:**
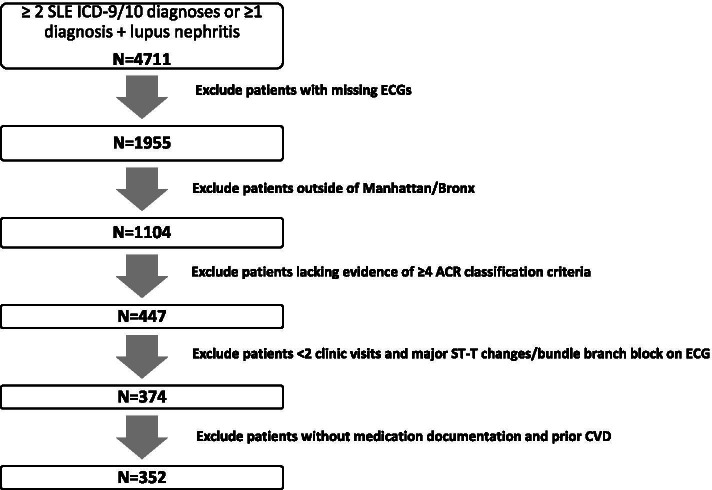
Columbia University Lupus Cohort

With approval from the Columbia University Institutional Review Board, electronic clinical data from the Columbia University Lupus Cohort registry was retrospectively reviewed. The patients consisted of all inpatients and outpatients seen at the Columbia University Irving Medical Center (CUIMC)/New York Presbyterian Hospitals (NYPH) with an SLE or lupus nephritis diagnosis attested by the International Classification of Diseases, Ninth and Tenth Revision, Clinical Modification (ICD-9/ICD-10) billing code diagnosis, between January 2015 and December 2019. Inclusion criteria were as follows: 1) ≥ 2 SLE ICD-9/ICD-10 billing diagnoses confirmed by manual chart review (fulfilling at least 4 ACR classification criteria [15]) or ≥ 1 SLE diagnosis PLUS ≥ 1 lupus nephritis proven on renal pathology report review; 2) at least 2 clinical visits on record; 3) ECG information available; and 4) those residing locally in the boroughs of Manhattan and Bronx (to increase the likelihood of having patients with continuous and multidisciplinary care at CUIMC). Exclusion criteria were as follows: 1) major ST-T changes/bundle branch block on ECG, 2) prior CVD, and 3) missing documentation of medications. These criteria are summarized in Fig. 1.

### RA cohorts

Two established RA cohorts were studied: 1) *ESCAPE-RA* (Evaluation of Subclinical Cardiovascular Disease and Predictors of Events in Rheumatoid Arthritis) was a prospective study to investigate subclinical atherosclerosis in an RA cohort without clinical CV disease [16]. Participants were recruited from the Johns Hopkins Arthritis Center and referrals from community rheumatologists between 2004 and 2008. Inclusion criteria were age > 45 for men and > 50 for women and fulfillment of the 1987 ACR RA classification criteria [17]. 2) *RHYTHM-RA* (RHeumatoid arthritis: studY of The Myocardium) is a cross-sectional study (subsequently extended to 4–6-year follow-up) of myocardial phenotypes in RA patients without clinical CVD recruited from CUIMC and local rheumatology clinics between 2011 and 2020. Inclusion criteria included age ≥ 18 years old and fulfillment of 2010 ACR RA classification criteria [15]. In both RA cohorts, ECGs were obtained during the first study visit.

### Outcome measure

#### QTc length

The 12-lead ECGs (25 mm/s paper speed and 10 mm/mV amplitude) obtained at the first/baseline study visits (RA) and regular clinical care (SLE) were interpreted by a board-certified cardiologist (PP) with specialization in electrophysiology blinded to diagnosis. The QT-interval was calculated and adjusted for the heart rate using Bazett’s formula (QTc = QT/√RR) [18] and evaluated both as a continuous variable and as a binary variable using cutoffs of ≥ 440 and ≥ 500 ms. These cutoffs have been associated with an increased risk of clinical cardiac events including myocardial infarction, cardiac arrest, and stroke, as well as sudden cardiac death [19–21].

### Clinical covariates

#### SLE

Patient characteristics and medications were collected from chart review. SLE disease duration was calculated as the duration in years from the date of physician diagnosis. Medication data were ascertained via clinician notes from the Electronic Medical Record (EMR). All medication data including HCQ use were ascertained at the time of the ECG.

#### RA cohorts (ESCAPE-RA/RHYTHM)

Patient characteristics and medications were obtained through study patient questionnaires. RA disease duration was assessed by patient self-report of the date of diagnosis. Medication data was ascertained from medication bottles, and HCQ use was ascertained at the time of the ECG. Information regarding cumulative dosage or length of therapy for HCQ were not available for both SLE and RA cohorts. Hypertension was defined as a systolic blood pressure (BP) of ≥ 140 mm Hg or diastolic BP of ≥ 90 mm Hg or use of antihypertensives at the time of the evaluation. Diabetes was defined as a fasting serum glucose of ≥ 126 mg/dL or glycosylated hemoglobin (HbA1c) greater than 6.4% or antidiabetic medication use. QT-modifying medications were defined as any medication included in the following categories: antidepressants, antipsychotics, antiarrhythmics, muscle relaxants, antimicrobials (antivirals/macrolides/fluoroquinolones), tacrolimus, anticonvulsants, and antiemetics. Medication data (clearly noted as taking in EMR or study visits) was recorded in 76% of biologics, 92% of steroids, 77% of statin, 79% of aspirin, and 38% of any QTc prolonging medications. If QTc prolonging medication use (categorical variable) was not reported on the medication list, it was assumed that the patient was not on QTc prolonging medications.

### Laboratory covariates

#### SLE

Laboratory measures, including anti-cardiolipin IgG/IgM (acL IgG/IgM), anti-nuclear antigen (ANA), anti-extra nuclear antigen (anti-ENA), double strand DNA antibody, lupus anticoagulant (LAC), anti-Sjogren's syndrome type a/b antibody (SSA/SSB), anti-Smith, U1 small nuclear ribonucleoprotein antibody (U1-RNP), C-reactive protein (CRP), and complement levels (C3, C4) were collected from the EMR.

#### RA cohorts (ESCAPE-RA/RHYTHM)

Rheumatoid factor (seropositivity ≥ 40 units; IBL America, Minneapolis, MN) and anti-CCP (anticyclic citrullinated peptide antibody) (seropositivity ≥60 units; Inova Diagnostics, Woburn, MA) were measured using enzyme-linked immunosorbent assay (ELISA). The levels of C-reactive protein (CRP) were measured in the Biomarkers Core Laboratory of the CUIMC Clinical and Translational Research Center.

### Statistical analysis

Variables were summarized and compared using Student’s *t* tests if normally distributed, Wilcoxon rank-sum tests for non-normally distributed variables, or *χ*^2^ or Fisher’s exact tests for categorical variables. Linear and logistic regression were used to model the associations of clinical and laboratory covariates with QTc length (continuous), and with QTc ≥440 ms and QTc ≥ 500 ms, respectively. Multivariable models were constructed by including any covariate significantly (*p* < 0.25) associated with the primary outcomes (QTc length, QTc ≥ 440 ms, QTc ≥ 500 ms) in univariate regression models. All analyses were performed using Stata version 15 (StataCorp, College Station, TX).

Multiple chained imputations were used to impute medication use for those with missing data for medications associated with QTc length (continuous) and QTc ≥ 440 and ≥ 500.

## Results

### Patient population

Patient disease characteristics, medications, and CV risk factors of combined SLE and RA cohorts are summarized in Table 1. Of the 530 study patients included in the study, 371 (70%) reported HCQ use at the time of ECG assessment. In the combined SLE/RA cohort, the mean QTc was 437 ± 29 ms. The mean QTc measurements for the SLE and RA cohorts were 432 ± 23 ms and 444 ± 33 ms, respectively. Forty-four percent of the combined group had a QTc ≥ 440 ms, and 7% had a QTc ≥ 500 ms. On average, the cohort was middle aged (51 ± 14 years), predominantly female (83%), and non-white race (combined Black, Hispanic, and other race 62%). The median disease duration was 12 years, and 65% were on glucocorticoids. Hypertension was reported in 46%, while 39% were on other QTc prolonging medications. On stratification by HCQ use, HCQ users were significantly younger and used more glucocorticoids and statins (*p < 0.005*) than non-HCQ users. No arrhythmic episodes or associated deaths were reported during the study periods for the RA or the SLE cohorts (2011–2020 and 2015–2019, respectively).

**Table 1:** Baseline characteristics SLE + RA (*N* = 530) stratified by HCQ use

Clinical characteristics	HCQ (***N*** = 371)	NO HCQ (***N*** = 159)	***p*** value
**Demographics**			
**Female** ***n*** **(%)**	329 (89)	136 (87)	0.51
**Age (mean ± SD)**	46.3(14.1)	55.3 (13.1)	***< 0.005***
**Race**			
**White** ***n*** **(%)**	83 (23)	57 (37)	***0.001***
**Black** ***n*** **(%)**	107 (29)	33 (21)	***0.052***
**Hispanic** ***n*** **(%)**	166 (46)	60 (38)	0.13
**Other** ***n*** **(%)**	8 (2)	6 (3.8)	0.29
**Disease characteristics**			
**Disease duration years (mean ± SD)**	12.4 (9.03)	11.5 (12.3)	0.43
**Immunosuppressant use**			
**Current biologics use** ***n*/total (%)**	67/238 (28.1)	59/159 (37.1)	0.060
**Current steroid use** ***n*/total (%)**	293/340 (86.2)	61/159 (38.4)	***< 0.005***
**CVD risk factors**			
**Hypertension** ***n*/total (%)**	170/369 (46.1)	76/155 (49)	0.54
**Diabetes mellitus** ***n*/total (%)**	25/368(7)	20/154(13)	***0.02***
**Current smoking** ***n*/total (%)**	23/371 (6.2)	15/158 (9.5)	0.18
**Current statin use** ***n*/total (%)**	79/244 (32.4)	30/159(18.9)	***0.003***
**Current aspirin use** ***n*/total (%)**	147/257 (57.2)	31/159(19.5)	***< 0.005***
**Ejection fraction% (mean ± SD)**	58.1 ± 9.4	62.2 ± 5.1	***< 0.005***
**Ejection fraction ≥ 55%** ***n*/total (%)**	337/371 (90.8)	150/159 (93.3)	0.18
**QTc average (mean ± SD)**	434 ± 25.7	441 ± 30.3	***0.0079***
**QTc ≥ 440** ***n*** **(%)**	134/371 (36.1)	86/159 (54.1)	***< 0.005***
**QTc ≥ 500** ***n*** **(%)**	13/371 (3.5)	23/159 (14.5)	***< 0.005***
**QTc Meds**			
**Any QTc meds** ***n*/total (%)**	170/368 (46.2)	52/148 (35.1)	***0.022***
**Antidepressants** ***n*/total (%)**	61/173 (35.3)	32/44 (72.7)	***< 0.005***
**Antipsychotics** ***n*/total (%)**	15/150 (10)	4/18 (22.2)	0.13
**Antiarrhythmics** ***n*/total (%)**	3/145 (2.07)	8/25 (32)	< 0.01
**Muscle relaxants** ***n*/total (%)**	11/148 (7.4)	5/22 (22.7)	***0.038***
**Antimicrobials** ***n*/total (%)**	83/157 (52.9)	12/20 (60)	0.55
**Tacrolimus** ***n*/total (%)**	26/155 (16.77)	1/17 (5.9)	0.21
**Anticonvulsants** ***n*/total (%)**	17/147(11.6)	1/18 (5.6)	0.39
**Antiemetics** ***n*/total (%)**	54/149 (36.2)	11/20 (55%)	0.10

*Abbreviations*: *CVD* cardiovascular disease, *HCQ* hydroxychloroquine, *QTc* QT corrected interval, *SD* standard deviation

### Association between HCQ use and QTc

**Fig. 2:**
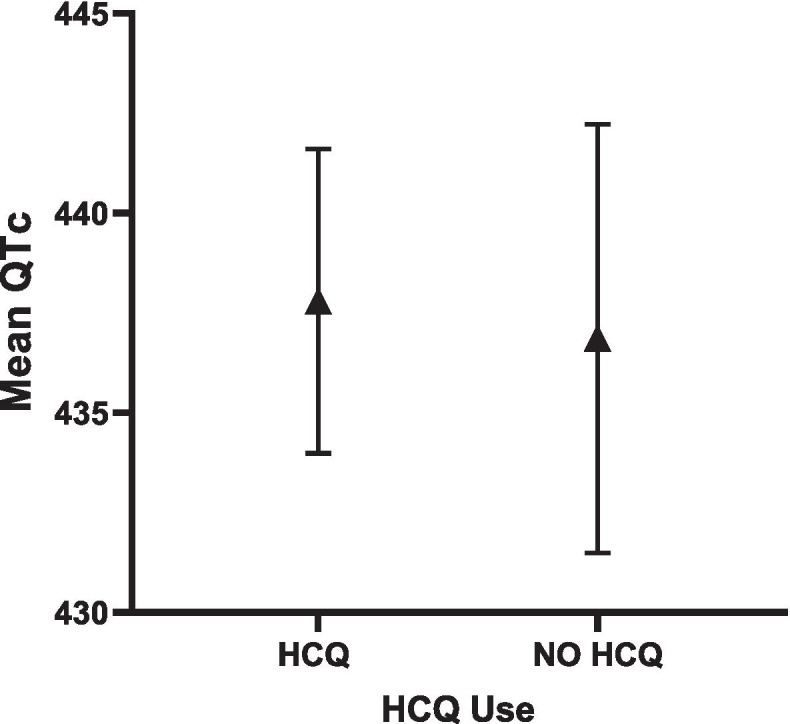
Adjusted QTc length and 95% CI in HCQ vs. NO HCQ in combined SLE/RA cohorts

**Fig. 3:**
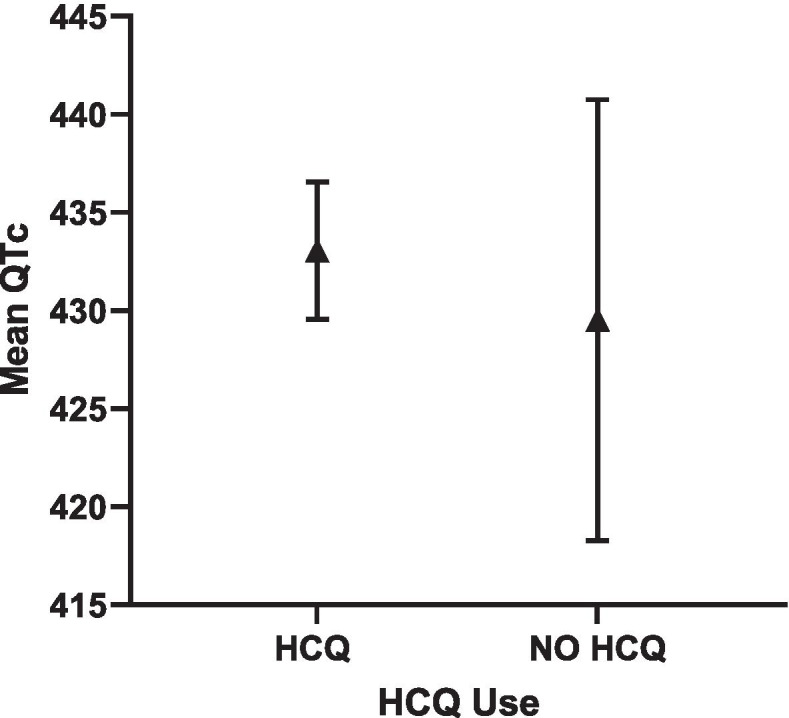
Adjusted QTc length and 95% CI in HCQ vs. NO HCQ in SLE cohort

**Fig. 4:**
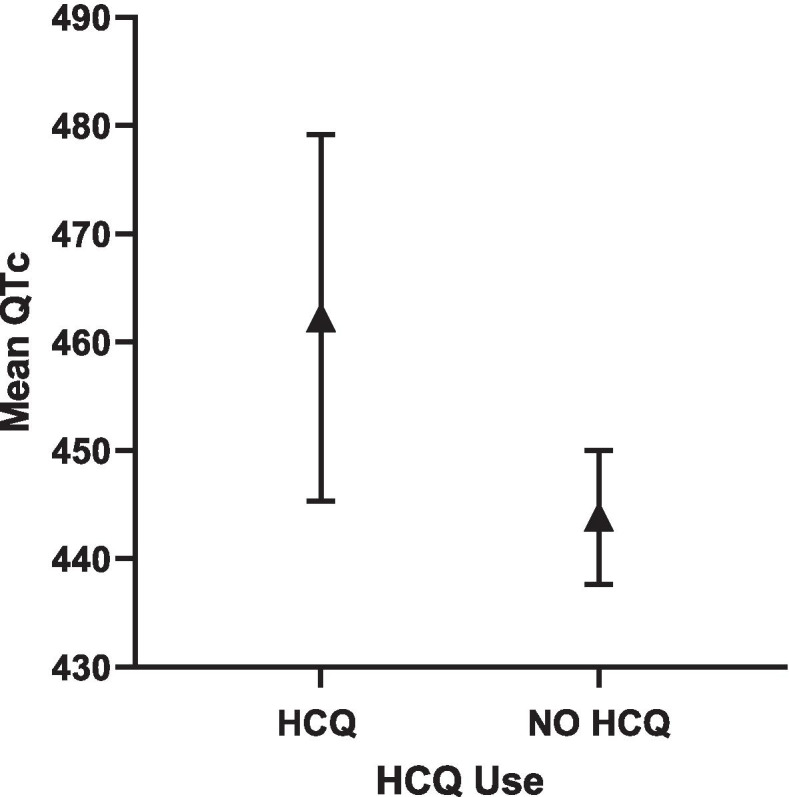
Adjusted QTc length and 95% CI in HCQ vs. NO HCQ in RA cohort

In an adjusted multivariable model, current use of HCQ was not significantly associated with mean QTc length (Table 2), as the mean adjusted QTc was 438 ms vs 437 ms for HCQ vs non-HCQ users, respectively) (Fig. 2). In the SLE only cohort, adjusted QTc was comparable in HCQ vs non-HCQ users (433 ms vs 427 ms, respectively) (Fig. 3). Similarly, in the RA only cohort, adjusted QTc was comparable in HCQ vs non-HCQ users (450 ms vs 443 ms) (Fig. 4). HCQ use was not a significant predictor of a QTc≥ 440 ms for the combined cohort (OR = 0.77; 95% CI 0.48–1.23, *p* = 0.27) (Table 3) in multivariable logistic models. Current HCQ use was inversely associated with QTc≥ 500 ms (OR = 0.39; 95% CI 0.16–0.98; *p = 0.044*) (Table 4).

**Table 2:** Associations of clinical characteristics with QTc length in combined SLE/RA cohort

Clinical covariates	Univariable model QTc (continuous)		Multivariable model QTc (continuous)^**a**^	
	***Β***	***p***	***Β***	***p***
**Age**	**0.38**	**< 0.005**	**0.21**	***0.033***
**Sex**	0.31	0.92	----------	----------
**Race (white vs non-white)**	6.2	**< 0.05**	− 0.29	0.92
**Disease duration (square root)**	− 0.1	0.86	----------	----------
**Current HCQ use**	− **7.8**	**< 0.005**	0.22	0.95
**Current prednisone use**	− 9.8	**< 0.005**	− **8.27**	***0.018***
**Current biologic use**	0.45	0.87	----------	----------
**Hypertension**	3.6	0.11	3.01	0.29
**Current smoking**	**10.6**	**< 0.05**	**10.2**	***0.033***
**Diabetes**	**8.9**	**< 0.05**	0.69	0.88
**Current statin use**	2.7	0.38	----------	----------
**Current aspirin use**	− 7.1	**< 0.05**	− 3.9	0.22
**Any QTc meds**	− 1.4	0.56	----------	----------
**Antidepressants**	**7.1**	**< 0.05**	----------	----------
**Antipsychotics**	− 1.6	0.80	----------	----------
**Antiarrhythmics**	5.4	0.49	----------	----------
**Muscle relaxants**	9.6	0.12	----------	----------
**Antimicrobials**	− 0.2	0.96	− 2.4	0.46
**Antiemetics**	1.8	0.63	----------	----------
**Anticonvulsants**	7.9	0.21	----------	----------
**Tacrolimus**	− 0.5	0.92	----------	----------
**Ejection fraction %**	0.05	0.77	----------	----------
**Ejection fraction ≥ 55%**	− 3.2	0.47	----------	----------
**Prob > F**	----------		***0.0001***	

*Abbreviations*: *HCQ* hydroxychloroquine, *QtC* QT corrected interval

^a^Final imputed data

**Table 3:** Associations of clinical characteristics with QTc ≥ 440 ms in combined SLE/RA cohort

Clinical covariates	Univariable model QTc ≥ 440 ms			Multivariable model QTc ≥ 440 ms^**b**^		
	***OR***	***95% CI***	***p***	***OR***	***95% CI***	***p***
**Age**	**1.02**	**1.00–1.03**	**0.004**	1.00	0.99**–**1.02	0.53
**Sex**	1.29	0.86**–**1.96	0.22	----------	----------	----------
**Race (white vs non-white)**	**1.48**	**1.07–2.03**	**0.016**	1.06	0.68**–**1.63	0.80
**Disease duration (square root)**	0.96	0.86**–**1.07	0.48	----------	----------	----------
**Current HCQ use**	**0.48**	**0.33–0.70**	**< 0.01**	0.77	0.48**–**1.23	0.27
**Current prednisone use**	**0.46**	**0.33–0.64**	**< 0.01**	**0.56**	**0.34–0.92**	***0.023***
**Current biologic use**	0.83	0.58**–**1.19	0.30	----------	----------	----------
**Hypertension**	1.18	0.87**–**1.61	0.29	----------	----------	----------
**Current smoking**	1.46	0.82**–**2.60	0.20	----------	----------	----------
**Diabetes**	**1.64**	**0.95–2.83**	**0.076**	1.38	0.70**–**2.72	0.35
**Current statin use**	1.08	0.73**–**1.60	0.69	----------	----------	----------
**Current ASA use**	**0.51**	**0.36–0.73**	**< 0.01**	**0.68**	**0.43–1.07**	***0.095***
**Any QTc meds**	1.07	0.78**–**1.47	0.67	----------	----------	----------
**Antidepressants**	**1.79**	**1.07–2.99**	**0.027**	1.45	0.90**–**2.33	0.12
**Antipsychotics**	1.62	0.62**–**4.22	0.33	----------	----------	----------
**Antiarrhythmics**	1.48	0.43**–**5.06	0.53	----------	----------	----------
**Muscle relaxants**	1.41	0.57**–**3.46	0.46	----------	----------	----------
**Antimicrobials**	0.99	0.62**–**1.58	0.97	----------	----------	----------
**Antiemetics**	1.15	0.63**–**2.08	0.65	----------	----------	----------
**Anticonvulsants**	2.33	0.54**–**1.93	0.94	----------	----------	----------
**Tacrolimus**	1.63	0.72**–**3.69	0.24	----------	----------	----------
**Ejection fraction %**	1.00	0.98**–**1.03	0.82	----------	----------	----------
**Ejection fraction ≥ 55%**	0.81	0.44**–**1.49	0.50	----------	----------	----------
**Prob > F**	----------			***0.0001***		

*Abbreviations*: *HCQ* hydroxychloroquine, *QTcMeds* QTc prolonging medications

^b^Final imputed data

**Table 4:** Associations of clinical characteristics with QTc ≥ 500 ms in combined SLE/RA cohort

Clinical covariates	Univariable model QTc ≥ 500 ms			Multivariable model QTc ≥ 500 ms^**c**^		
	***OR***	***95% CI***	***p***	***OR***	***95% CI***	***p***
**Age**	0.99	0.96**–**1.01	0.25	----------	----------	----------
**Sex**	1.12	0.49**–**2.57	0.79	----------	----------	----------
**Race (white vs non-white)**	0.92	0.48**–**1.76	0.79	----------	----------	----------
**Disease duration (square root)**	0.86	0.69**–**1.07	0.17	----------	----------	----------
**Current HCQ use**	**0.21**	**0.11–0.44**	**< 0.01**	**0.39**	**0.16–0.98**	***0.044***
**Current prednisone use**	**0.43**	**0.23–0.81**	**0.010**	0.55	0.23**–**1.34	0.19
**Current biologic use**	0.94	0.48**–**1.85	0.86	----------	----------	----------
**Hypertension**	1.37	0.74**–**2.55	0.31	----------	----------	----------
**Current smoking**	**2.71**	**1.13–6.45**	**0.025**	2.23	0.80**–**6.15	0.12
**Diabetes**	**2.73**	**1.20–6.23**	**0.017**	1.47	0.47**–**4.63	0.51
**Current statin use**	0.56	0.24**–**1.29	0.17	----------	----------	----------
**Current aspirin use**	**0.39**	**0.18–0.83**	**0.015**	0.60	0.23**–**1.56	0.29
**Any QTc meds**	1.14	0.63**–**2.07	0.66	----------	----------	----------
**Antidepressants**	**2.41**	**0.88–6.56**	**0.085**	1.30	0.52**–**3.23	0.58
**Antipsychotics**	1.14	0.13**–**9.83	0.90	----------	----------	----------
**Antiarrhythmics**	2.20	0.25**–**19.67	0.48	----------	----------	----------
**Muscle relaxants**	**3.36**	**0.80–14.10**	**0.098**	3.04	0.68**–**13.63	0.15
**Antimicrobials**	0.55	0.15**–**2.01	0.36	----------	----------	----------
**Antiemetics**	1.0	----------	----------	----------	----------	----------
**Anticonvulsants**	1.19	0.14**–**10.29	0.87	----------	----------	----------
**Tacrolimus**	**4.29**	**0.90–20.33**	**0.067**	**4.31**	**1.06–17.53**	***0.042***
**Ejection fraction %**	**1.07**	**0.99–1.15**	**0.060**	1.05	0.96**–**1.14	0.31
**Ejection fraction ≥ 55%**	1.79	0.42**–**7.63	0.43	----------	----------	----------
**Prob > F**	----------			***0.0066***		

*Abbreviations*: *HCQ* hydroxychloroquine, *QTcMeds* QTc prolonging medications

^c^Final imputed data

### Significant associations with QTc length

In multivariable models, age, current prednisone use, and current smoking were significantly associated with QTc length (Table 2). Current prednisone use and current aspirin use were significantly associated with QTc≥ 440 ms (Table 3). The only significant predictor of QTc≥ 500 ms was current use of tacrolimus (Table 4).

### Subgroup analyses

#### SLE only cohort

In the SLE cohort, HCQ use was not a significant predictor of QTc length (Supplementary Table 3) or a QTc ≥ 440 ms (Supplementary Table 4). However, of the 11 SLE patients with QTc ≥ 500 ms, 9/11 were reported to be on HCQ. Given the small sample size, statistically significant differences could not be ascertained between the HCQ groups with a QTc ≥500 ms, yet no arrhythmias or associated deaths were reported, as per retrospective chart review over 4 years. In multivariable analyses, elevated CRP level (≥ 10.0 mg/L) was significantly associated with QTc length, and diabetes and use of any QTc prolonging medications were significantly associated with QTc ≥ 440 ms (Supplementary Table 4). Adjusted QTc was comparable in the HCQ vs non-HCQ users groups (433 ms vs 429 ms, respectively) (Fig. 3).

#### RA only cohorts

In the RA cohorts analyzed separately, HCQ use was a significant predictor of QTc length (Supplementary Table 6), and adjusted QTc was longer in HCQ vs non-HCQ users (462 ms vs 443 ms, respectively) (Fig. 4). However, HCQ use was not a predictor of QTc ≥ 440 (*p* = 0.79) or ≥500 ms (*p* = 0.41) (Supplementary Tables 7 and 8). Notably, significant predictors of prolonged QTc≥ 500 ms included age, current smoking, and diabetes (Supplementary Table 8).

#### Interaction with other QTc prolonging medications

**Fig. 5:**
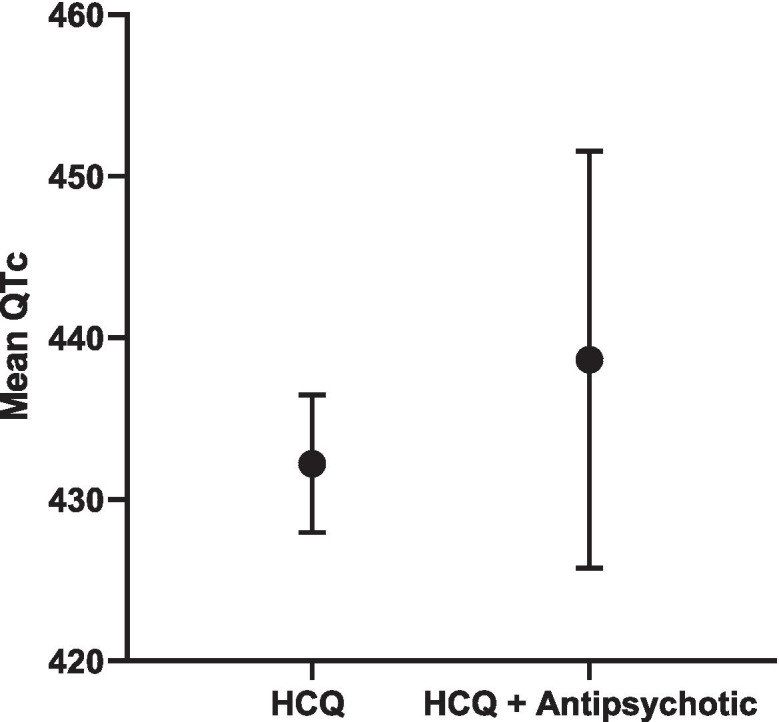
Mean QTc length and 95% CI in HCQ vs. HCQ + antipsychotic in SLE/RA cohort

**Fig. 6:**
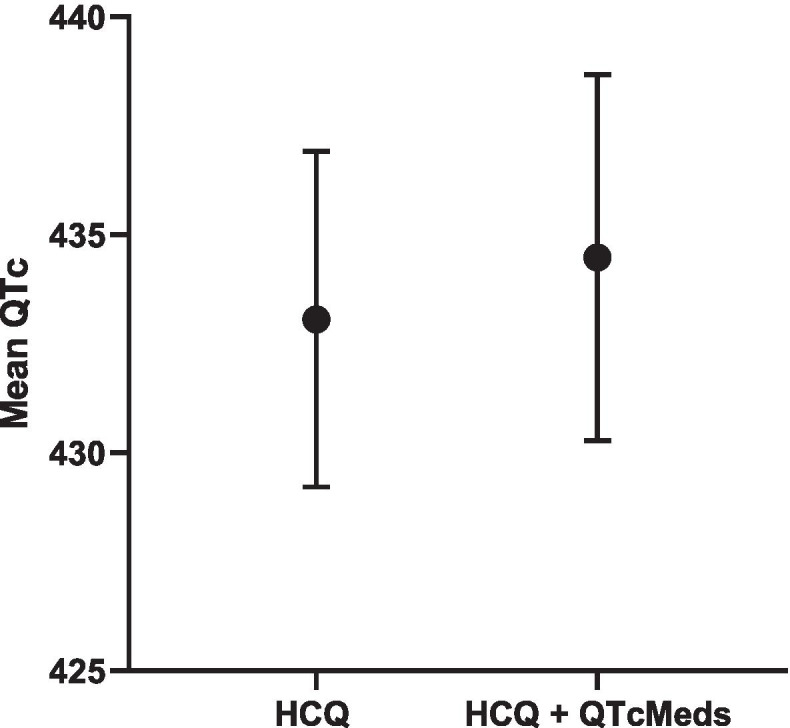
Mean QTc length and 95% CI in HCQ vs. HCQ+QTcMeds in combined SLE/RA cohort

In the combined RA + SLE cohort, a significant interaction was found between HCQ use and use of anti-psychotics (Table 5), with QTc length being longer in those on both vs only HCQ (439 ms vs 432 ms; Fig. 5). Overall, QTc length was comparable in those on HCQ + any QTc prolonging medications vs only HCQ (434 ms vs 433 ms; Fig. 6). When stratified by RA vs SLE cohort, no significant interactions were found between HCQ use and use of any QTc medications (Supplementary Table 9–10).

**Table 5:** Interactions in combined SLE/RA cohort

QTc	***Β***	***p***
**Current HCQ Use#AnyQTcMeds**	3.70	0.52
**Current HCQ Use#Antidepressants**	4.35	0.65
**Current HCQ Use#Antipsychotics**	**40.1**	***0.01***
**Current HCQ Use#Antiarrhythmics**	7.46	0.68
**Current HCQ Use#Musclerelaxants**	− 2.95	0.89
**Current HCQ Use#Antimicrobials**	− 1.47	0.90
**Current HCQ Use#Antiemetics**	2.97	0.80
**Current HCQ Use#Tacrolimus**	22.23	0.39

## Discussion

In this large cohort of RA and SLE patients, HCQ use was not associated with QTc length when adjusted for potential confounders such as age and other medications affecting QTc length. The adjusted QTc length was comparable between HCQ users and non-users (438 ms vs 437 ms). Although up to 44% of the combined cohort had a QTc ≥ 440 ms, HCQ use was not a predictor of prolonged QTc.

QTc length as an outcome remains of paramount interest, since in the general population and in selected subpopulations (i.e., the elderly, patients with coronary artery disease, and the critically ill), prolonged QTc length (defined in those studies as > 450 ms in men and > 470 ms in women) independently predicts sudden cardiac death [19, 20]. In fact, even moderate QTc prolongation between 420 and 440 ms has been associated with all-cause mortality [21]. In a retrospective cohort study of RA patients, idiopathic QTc prolongation [22] was associated with an almost 30% increase in all-cause mortality (HR 1.28; 95% CI 0.91–1.81, *p* = 0.16). Furthermore, in a prospective cohort of RA patients, a 50-ms increase in QTc interval was independently associated with a twofold risk of mortality (HR =2.18, 95% CI 1.09, 4.35), and CRP levels were also independently associated with QTc length [10]. In our study, prednisone use was associated with a lower QTc length in the combined RA + SLE cohort, in addition to being a negative predictor of QTc≥440 ms. From this observation, we hypothesize that reduction of systemic inflammation mitigated any deleterious effects on the conduction system and thus decreased the risk of QTc prolongation. However, when we examined these associations separately by individual cohorts, in the SLE cohort, CRP > 10 mg/L and not prednisone use was associated with (higher) QTc length. Given that 79% of our SLE cohort demonstrated CRP > 10 mg/L, despite 93% using prednisone, we postulate that in this group, high disease activity (and thus inflammatory) burden, unmitigated by prednisone use, increased the risk of QTc prolongation. The specific interplay between inflammation and arrhythmogenic potential, as well as mechanisms leading to arrhythmia, merits further exploration.

HCQ-associated QTc prolongation and subsequent arrhythmia development received considerable attention during its widespread use in COVID-19 patients. In an uncontrolled study of COVID-19 patients receiving HCQ alone or HCQ and azithromycin for associated pneumonia [8], baseline to treatment change in QTc was higher in the HCQ + azithromycin group vs HCQ alone. It is also worthwhile noting that in the prior study, up to 19% of those receiving HCQ alone had a QTc > 500 ms (and 21% in combination group) and 8% had a clinically significant increase > 60 ms, with one episode of torsades de pointes reported. However, independent effects of COVID-19 infection on the cardiac conduction system [9] and indication bias (severe COVID-19 patients more likely to receive HCQ) must be considered in interpretation of these data. Similarly, Ramireddy et al. [23] reported a significantly higher change in QTc from baseline to treatment in the HCQ and azithromycin group vs HCQ alone (17 ± 39 ms vs 0.5 ± 40 ms; *p* = *0.07*). More concerning, up to 12% of total patients in this study (receiving HCQ alone, azithromycin alone, or both) had critical QTc prolongation (defined as maximum QTc ≥ 500 ms (if QRS < 120 ms) or QTc ≥ 550 ms (if QRS ≥120 ms) and QTc increase of ≥ 60 ms); however, no torsades de pointes was documented. The results of a more recent randomized controlled trial [24] were more reassuring in that, in 1561 COVID patients randomized to the HCQ arm and loaded with high doses of HCQ (800 mg × 2 doses followed by 400 mg every 12 h for 9 days or until discharge), there were no significant differences in terms of frequency of arrhythmias compared to the usual care group.

As for rheumatologic patients, SLE patients treated with high cumulative doses (700–1300 g) of antimalarials from several months to decades demonstrated bundle branch block and third-degree AV block (with some leading to Torsades de Pointes) [25–28]. However, interpretation from these case reports is limited due to absence of controls. It is also important to note that current trends in HCQ dosing have become more conservative due to heightened awareness of retinal toxicity. More recently, Lane et al. [29] reported *no increased risk* of cardiac arrhythmias (calibrated HR 0.90; 95% CI 0.78–1.03; *p < 0.01*) in HCQ users (400 mg/day for 30 days) vs sulfasalazine users in a retrospective review of 14 multinational databases of RA patients. Liu et al. [30] reported a *lower* risk of CV disease including sudden cardiac arrest/death in HCQ/chloroquine (CQ) users vs non-users (RR 0.72; 95% CI 0.56–0.94; *p = 0.013*) in a meta-analysis of various rheumatologic patients. Various cardioprotective (thromboprotective and cholesterol reducing) effects of HCQ/CQ [31, 32] may partially explain this finding but the absence of clinical trial data and CV/metabolic parameters limit interpretation. In another prospective study [33] of RA patients, incidence of long QT syndrome or arrhythmia-related hospitalizations were comparable between HCQ use vs non-HCQ disease-modifying anti-rheumatic drug (DMARD) use.

Specifically, the lack of association of HCQ use with overall QTc length in our results is consistent with prior publications in RA and SLE patients [10–13, 22]. The main strength of our study is its sample size as it represents one of the largest multiethnic studies inclusive of both SLE and RA patients. Importantly, we accounted for the concurrent use of a wide variety of QTc prolonging or arrhythmogenic medications, which was not consistently done in previous literature. Although SLE data were obtained retrospectively via ICD 9/10 codes on EMR review, we restricted analyses to SLE patients who demonstrated consistent care at our institution (≥ 2 visits). For both the SLE and RA cohorts, QTc length was calculated by standardized Bazett’s formula and confirmed by a blinded, trained cardiac electrophysiologist (PP). The main limitations of our study include the lack of data on HCQ adherence (i.e., via patient report, and/or metabolite levels), as well as cumulative dosage or length of therapy, as it is known that HCQ adherence in the SLE population is variably poor and the risk of HCQ toxicity increases with cumulative dosage. Moreover, although we detected a statistically significant interaction between antipsychotics and HCQ use, we acknowledge the small number of observations (< 20 who reported on both antipsychotic and HCQ use) that may preclude interaction testing as well as produce an overestimation of interaction effect. In addition, we did not obtain or analyze pre-HCQ ECGs (determined only at the time of HCQ use for both SLE and RA cohorts) and therefore cannot make conclusions about pre- and post-exposure change in QTc length. Finally, because we excluded patients with clinical CVD who received ECG screening, our findings may not directly apply to those patients, who may have other risk factors associated with prolonged QTc. Given the prevalence of associated CV risk factors (hypertension, diabetes), we surmise that our patients likely had some underlying, subclinical CVD.

## Conclusions

In a combined large multiethnic cohort of RA and SLE patients, QTc length did not significantly differ in HCQ users compared with non-HCQ users, nor was it associated with a QTc ≥ 440 ms, even while adjusting for potential confounders. There was a notable statistical interaction between the use of HCQ and use of antipsychotics in the combined RA and SLE cohort. Our data suggests that HCQ does not increase the arrhythmogenic risk for patients with rheumatologic conditions.

## Supplementary Information

**Additional file 1: Table 1**. Baseline Characteristics SLE (N=352) Stratified by HCQ Use. **Table 2**. Baseline Characteristics RA (N=178) Stratified by HCQ Use. **Table 3**. Associations of Clinical Characteristics with QTc Length in SLE Cohort. **Table 4**. Associations of Clinical Characteristics with QTc≥440 ms in SLE Cohort. **Table 5**. Associations of Clinical Characteristics with QTc≥500 ms in SLE Cohort. **Table 6**. Associations of Clinical Characteristics with QTc Length in RA Cohort. **Table 7**. Associations of Clinical Characteristics with QTc≥440 ms in RA Cohort. **Table 8**. Associations of Clinical Characteristics with QTc≥500 ms in RA Cohort. **Table 9**. Interactions in RA Cohort. **Table 10**. Interactions in SLE Cohort.

## Data Availability

The datasets used and analyzed during the current study are included in this published article (and its supplementary information files) or otherwise available from the corresponding author on request.

## Abbreviations

AcL IgG/IgM: Anti-cardiolipin IgG/IgM
ACR: American College of Rheumatology
ANA: Anti-nuclear antigen
Anti-CCP: Anticyclic citrullinated peptide antibody
Anti-ENA: Anti-extra nuclear antigen
BP: Blood pressure
COVID-19: Coronavirus disease 19
CQ: Chloroquine
CRP: C-reactive protein
CUIMC: Columbia University Irving Medical Center
CV/D: Cardiovascular disease
DMARD: Disease-modifying anti-rheumatic drug
ECG: Electrocardiograms
ELISA: Enzyme-linked immunosorbent assay
EMR: Electronic Medical Record
ESCAPE-RA: Evaluation of Subclinical Cardiovascular Disease and Predictors of Events in Rheumatoid Arthritis
HbA1c: Glycosylated hemoglobin
HCQ: Hydroxychloroquine
ICD-9/ICD-10: International Classification of Diseases, Ninth and Tenth Revision, Clinical Modification
LAC: Lupus anticoagulant
NYPH: New York Presbyterian Hospitals
QTc: Corrected QT length
RA: Rheumatoid arthritis
RHYTHM-RA: RHeumatoid arthritis: studY of The Myocardium
SLE: Systemic lupus erythematosus
SSA/SSB: Anti-Sjogren’s syndrome type a/b antibody
U1-RNP: U1 small nuclear ribonucleoprotein
