# Supplementary material for: Hydroxychloroquine use is not associated with QTc length in a large cohort of SLE and RA patients
Source: Arthritis Res Ther. 2021 Oct 29;23:271. doi: 10.1186/s13075-021-02646-0 (PMC8554185; doi:10.1186/s13075-021-02646-0)
Supplement: Supplementary file 1 — Additional file 1: Table 1. Baseline Characteristics SLE (N=352) Stratified by HCQ Use. Table 2. Baseline Characteristics RA (N=178) Stratified by HCQ Use. Table 3. Associations of Clinical Characteristics with QTc Length in SLE Cohort. Table 4. Associations of Clinical Characteristics with QTc≥440 ms in SLE Cohort. Table 5. Associations of Clinical Characteristics with QTc≥500 ms in SLE Cohort. Table 6. Associations of Clinical Characteristics with QTc Length in RA Cohort. Table 7. Associations of Clinical Characteristics with QTc≥440 ms in RA Cohort. Table 8. Associations of Clinical Characteristics with QTc≥500 ms in RA Cohort. Table 9. Interactions in RA Cohort. Table 10. Interactions in SLE Cohort. [file 13075_2021_2646_MOESM1_ESM.docx]

**Supplementary Section**

**Table1. Baseline Characteristics SLE (N=352) Stratified by HCQ Use**

| **Clinical Characteristics** | **HCQ (N=329)** | **NO HCQ (N=23)** | **p-value** |
| --- | --- | --- | --- |
| **Demographics** |  |  |  |
| **Female n (%)** | 297 (90.3%) | 22 (96.6%) | 0.71 |
| **Age (Mean ± SD)** | 45.0±13.9 | 63.7±15.3 | ***<0.05*** |
| **Race** |  |  |  |
| **White n (%)** | 55 (17.0%) | 7 (30.4%) | 0.10 |
| **Black n (%)** | 106 (32.8%) | 6 (26.1%) | 0.50 |
| **Hispanic n (%)** | 156 (48.3%) | 10 (43.5%) | 0.63 |
| **Other n (%)** | 5 (1.55%) | 0 | ------ |
| **Disease Characteristics** |  |  |  |
| **Disease duration years (Mean ± SD)** | 12.8+9.2 | 17.7+10.5 | ***0.021*** |
| **APS n (%)** | 24 (16.4%) | 4 (22.2%) | 0.54 |
| **aCL IgM n (%)** | 84(33.7%) | 8 (40%) | 0.57 |
| **aCL IgG n (%)** | 83 (32.9%) | 8 (40%) | 0.52 |
| **LN dx n (%)** | 118 (43.1%) | 12 (57.1%) | 0.21 |
| **DNA n (%)** | 234 (79.0%) | 15 (68.2%) | 0.23 |
| **Low c3/c4 n (%)** | 202(78.3%) | 8 (40%) | ***<0.005*** |
| **CRP n (%)** | 219 (78.5%) | 18 (81.8%) | 0.71 |
| **ANA n (%)** | 299 (97.4%) | 20 (86.9%) | ***0.007*** |
| **Anti-ENA n (%)** | 108(61.0%) | 11 (55%) | 0.60 |
| **U1RNP n (%)** | 132 (51.9%) | 6 (31.6%) | 0.086 |
| **Smith n (%)** | 110(42.5%) | 4 (21.0%) | 0.067 |
| **DNA n (%)** | 234 (79.0%) | 15 (68.2%) | 0.23 |
| **LAC n (%)** | 57 (28.2%) | 5 (25%) | 0.76 |
| **SSA n (%)** | 141 (54.0%) | 8 (24.1%) | 0.31 |
| **SSB n (%)** | 57 (23.3%) | 2 (10.5%) | 0.19 |
| **Anti-CCP n (%)** | 14 (9.15%) | 0 | 0.37 |
| **Immunosuppressants** |  |  |  |
| **Current biologic use n (%)** | 53 (27.0%) | 3 (13.0%) | 0.145 |
| **Cyclophosphamide n (%)** | 64 (31.4%) | 4(17.4%) | 0.16 |
| **MMF n (%)** | 163 (67.4%) | 8 (34.8%) | ***0.002*** |
| **AZA n (%)** | 86 (39.3%) | 4 (17.4%) | ***0.039*** |
| **Current prednisone use n (%)** | 281 (94.3%) | 16 (69.6%) | ***<0.005*** |
| **DMARDs (leflunomide) n (%)** | 35 (18.3%) | 1 (4.3%) | 0.09 |
| **CVD Risk Factors** |  |  |  |
| **Hypertension n (%)** | 157 (47.7%) | 19 (82.6%) | ***0.001*** |
| **Diabetes Mellitus n (%)** | 23 (7.0%) | 3 (13.0%) | 0.29 |
| **Smoking n (%)** | 310 (94.2%) | 1 (4.3%) | 0.77 |
| **Current statin use n (%)** | 75 (37.1%) | 10 (43.5%) | 0.55 |
| **Current ASA use n (%)** | 136(63.3%) | 17 (73.9%) | 0.31 |
| **Ejection fraction % (Mean ± SD)** | 57.2 ±6.5 | 57.7±9.6 | 0.42 |
| **Ejection fraction≥55%** | 297 (90.3%) | 19(82.6%) | 0.24 |
| **QTc average (Mean ± SD)** | 431.7±23.9 | 434.6±23.2 | 0.57 |
| **QTc≥440 n (%)** | 109 (33.1%) | 8 (34.8%) | 0.87 |
| **QTc≥500 n (%)** | 9 (2.7%) | 0 | ----------- |
| **Any QTc meds n(%)** | 158 (48.2%) | 16 (69.6%) | ***0.047*** |
| **Antidepressants** | 51 (21.3%) | 6 (33.3%) | 0.86 |
| **Antipsychotics** | 13(8.8%) | 3(17.6%) | 0.22 |
| **Antiarrhythmics** | 3 (2.1%) | 0 | ----------- |
| **Muscle relaxants** | 8(5.5%) | 0 | ----------- |
| **Antimicrobials** | 82 (52.6%) | 9(52.9%) | 0.98 |
| **Tacrolimus** | 26(16.8%) | 1(5.9%) | 0.48 |
| **Anticonvulsants** | 17(11.6%) | 0 | 0.22 |
| **Antiemetics** | 53(35.8%) | 8(47.1%) | 0.36 |

**Abbreviations:** ***aCL IgG*, Anti-Cardiolipin IgG; *aCL IgM*, Anti-Cardiolipin IgM; *ANA*, Anti-Nuclear Antigen; *Anti-ENA*, Anti-Extra Nuclear Antigen; *APS*, Antiphospholipid Syndrome; *AZA*, Azathioprine; *Anti-CCP*, Cyclic Citrullinated Peptide Antibody; *CRP*, C-Reactive Protein; *DNA*, Double Strand DNA Antibody; *DMARDs*, Disease-Modifying Anti-rheumatic Drug; *HCQ*, Hydroxychloroquine; *LAC*, Lupus Anticoagulant; *LN dx*, Lupus Nephritis Diagnosis; *MMF*, Mycophenolate Mofetil; *QTcMeds*, QTc prolonging medications; *SSA*, Anti-Sjögren's Syndrome Type A Antibody; *SSB*, Anti-Sjögren's Syndrome Type B Antibody; *Sm*, Anti-Smith Antibody; *U1RNP*, U1 Small Nuclear Ribonucleoprotein Antibody**

**Table 2. Baseline Characteristics RA (N=178) Stratified by HCQ Use**

| **Clinical Characteristics** | **HCQ (N=42)** | **NO HCQ (N=136)** | **p-value** |
| --- | --- | --- | --- |
| **Demographics** |  |  |  |
| **Female, n (%)** | 32 (76.2%) | 114 (85.1%) | 0.18 |
| **Age (Mean ± SD)** | 56.7+9.7 | 53.9+12.3 | 0.18 |
| **Race** |  |  |  |
| **White** | 28 (68.29%) | 50 (37.59%) | ***0.001*** |
| **Black** | 1 (2.44%) | 27 (20.30%) | ***0.006*** |
| **Hispanic** | 9(21.95%) | 50(37.59%) | 0.064 |
| **Other** | 3(7.32%) | 6(4.51%) | 0.44 |
| **Disease Characteristics** |  |  |  |
| **Disease duration years (Mean ± SD)** | 9.6±7.5 | 10.5±12.3 | 0.39 |
| **RF n (%)** | 11(64.7%) | 115(89.1%) | ***0.006*** |
| **Anti-CCP n (%)** | 14 (82.3%) | 122(94.6%) | 0.094 |
| **CRP (Mean ± SD)** | 3.9±7.3 | 5.6±8.1 | 0.11 |
| **Immunosuppresants** |  |  |  |
| **Current biologic use n (%)** | 14 (33.3%) | 56 (41.2%) | 0.36 |
| **Current prednisone use n (%)** | 12 (28.6%) | 45 (33.1%) | 0.58 |
| **CVD Risk Factors** |  |  |  |
| **Hypertension n (%)** | 13(32.5%) | 57(43.2%) | 0.23 |
| **Diabetes mellitus n (%)** | 2 (5%) | 17(12.9%) | 0.25 |
| **Smoking n (%)** | 4(9.5%) | 14(10.4%) | 0.57 |
| **Current statin use n (%)** | 4 (9.5%) | 20 (14.7%) | 0.45 |
| **Current ASA use n (%)** | 11 (26.2%) | 14 (10.3%) | ***0.01*** |
| **Ejection fraction% (Mean ± SD)** | 62.8±5.1 | 62.7±4.8 | 0.89 |
| **Ejection fraction≥55% n(%)** | 40 (95.2%) | 131 (96.3%) | 0.75 |
| **QTc average (Mean ± SD)** | 450.0 ± 33.3 | 442.9±31.3 | 0.24 |
| **QTc≥440 n (%)** | 25 (59.5%) | 78 (57.3%) | 0.80 |
| **QTc≥500 n (%)** | 4(9.5%) | 23(16.9%) | 0.24 |
| **Any QTc meds n (%)** | 12 (30%) | 36(28.8%) | 0.88 |
| **Antidepressants** | 10 (23.8%) | 26 (19.1%) | 0.51 |
| **Antipsychotics** | 2(4.8%) | 1(0.74%) | 0.14 |
| **Antiarrhythmics** | 0 | 8(5.9%) | 0.20 |
| **Muscle relaxants** | 3(7.1%) | 5(3.7%) | 0.39 |
| **Antimicrobials** | 1(2.4%) | 3(2.2%) | ----------- |
| **Tacrolimus** | ----------- | ----------- | ----------- |
| **Anticonvulsants** | 0 | 1(0.74%) | ----------- |
| **Antiemetics** | 1(2.4%) | 3(2.2%) | ----------- |

**Abbreviations: *Anti-CCP*, Cyclic Citrullinated Peptide Antibody; *CRP*, C-Reactive Protein; *CVD*, Cardiovascular Disease; *QtC*, QT Corrected Interval; *RF*, Rheumatoid Factor**

**Table 3. Associations of Clinical Characteristics with QTc Length in SLE Cohort**

| **Clinical Covariates** | **Univariable Model QTc (Continuous)**  ***Β p*** | | **Multivariable Model QTc (Continuous)^d^**  ***Β p*** | |
| --- | --- | --- | --- | --- |
| **Age** | 0.21 | **0.012** | 0.077 | 0.46 |
| **Sex** | 3.62 | 0.387 | ---------- | ---------- |
| **Race (white vs non-white)** | -4.89 | 0.124 | ---------- | ---------- |
| **Disease duration years (square root)** | 1.12 | 0.229 | ---------- | ---------- |
| **LN dx** | 0.11 | 0.968 | ---------- | ---------- |
| **ANA** | -16.08 | **0.020** | **-13.93** | **0.074** |
| **DNA** | -6.17 | **0.053** | -4.27 | 0.22 |
| **Anti-ENA** | -5.98 | **0.072** | ---------- | ---------- |
| **U1RNP** | -5.51 | **0.040** | ---------- | ---------- |
| **SSA** | -4.97 | **0.068** | ---------- | ---------- |
| **SSB** | -7.21 | **0.029** | ---------- | ---------- |
| **Anti-CCP (binary)**  **≥19 units** | 2.90 | 0.648 | ---------- | ---------- |
| **CRP (binary)**  **≥10.0 mg/L** | 6.72 | **0.038** | **9.04** | **0.020** |
| **Current HCQ use** | -2.89 | 0.575 | 0.81 | 0.88 |
| **MMF** | -1.57 | 0.619 | ---------- | ---------- |
| **AZA** | 2.11 | 0.484 | ---------- | ---------- |
| **Current DMARDs use** | 1.41 | 0.742 | ---------- | ---------- |
| **Current prednisone use** | -2.25 | 0.663 | ---------- | ---------- |
| **Cyclophosphamide** | -1.43 | 0.677 | ---------- | ---------- |
| **Current biologic use** | -5.06 | 0.167 | ---------- | ---------- |
| **Smith** | -5.16 | **0.059** | -4.56 | 0.14 |
| **LAC** | 1.42 | 0.685 | ---------- | ---------- |
| **APS** | 1.19 | 0.806 | ---------- | ---------- |
| **aCL IgG** | -1.35 | 0.663 | ---------- | ---------- |
| **aCL IgM** | 0.58 | 0.850 | ---------- | ---------- |
| **Low c3/c4** | -5.23 | 0.110 | ---------- | ---------- |
| **Hypertension** | 3.50 | 0.157 | ---------- | ---------- |
| **Current smoking** | 9.74 | **0.072** | 8.49 | 0.15 |
| **Diabetes mellitus** | 12.72 | **0.005** | 5.82 | 0.27 |
| **Current statin use** | 9.43 | **0.007** | **6.29** | **0.078** |
| **Current aspirin use** | -0.47 | 0.89 | ---------- | ---------- |
| **Any QTc meds** | 3.44 | 0.16 | ---------- | ---------- |
| **Antidepressants** | 4.60 | 0.23 | ---------- | ---------- |
| **Antipsychotics** | 1.56 | 0.81 | ---------- | ---------- |
| **Antiarrhythmics** | 9.31 | 0.52 | ---------- | ---------- |
| **Musclerelaxants** | 1.32 | 0.88 | ---------- | ---------- |
| **Antimicrobials** | -1.11 | 0.76 | ---------- | ---------- |
| **Antiemetics** | 1.45 | 0.72 | ---------- | ---------- |
| **Anticonvulsants** | 7.87 | 0.21 | ---------- | ---------- |
| **Tacrolimus** | -0.54 | 0.92 | ---------- | ---------- |
| **Ejection fraction** | -0.25 | 0.15 | ---------- | ---------- |
| **Ejection fraction ≥55%** | **-6.81** | **0.095** | -2.82 | 0.52 |
| **Prob>F** | ---------- | | ***0.0023*** | |

**Abbreviations: *aCL IgG*, *Anti-Cardiolipin IgG*; *aCL IgM*, *Anti-Cardiolipin IgM*; *ANA*, Anti-Nuclear Antigen; *Anti-ENA*, Anti-Extra Nuclear Antigen; *APS*, Antiphospholipid Syndrome; *AZA*, Azathioprine; *Anti-CCP*, Cyclic Citrullinated Peptide Antibody; *CRP*, C-Reactive Protein; *DNA*, Double Strand DNA Antibody; *DMARDs*, Disease-Modifying Anti-rheumatic Drug; *HCQ*, Hydroxychloroquine; *LAC*, Lupus Anticoagulant; *LN dx*, Lupus Nephritis Diagnosis; *MMF*, Mycophenolate Mofetil; *QTcMeds*, QTc prolonging medications; *SSA*, Anti-Sjögren's Syndrome Type A Antibody; *SSB*, Anti-Sjögren's Syndrome Type B Antibody; *Sm*, Anti-Smith Antibody; *U1RNP*, U1 Small Nuclear Ribonucleoprotein Antibody**

**^d^represents final imputed data**

**Table 4. Associations of Clinical Characteristics with QTc≥440 ms in SLE Cohort**

| **Clinical Covariates** | **Univariable Model QTc≥440 ms**  ***OR 95% CI p*** | | | **Multivariable Model QTc≥440 ms^e^**      ***OR 95% CI p*** | | |
| --- | --- | --- | --- | --- | --- | --- |
| **Age** | 1.01 | 0.99-1.02 | 0.35 | ------ | --------- | ------ |
| **Sex** | 1.50 | 0.68-3.31 | 0.31 | ------ | ---------- | ------ |
| **Race (white vs non-white)** | 0.67 | 0.37-1.22 | 0.19 |  |  |  |
| **Disease duration years (square root)** | 1.04 | 0.89-1.23 | 0.60 | ------ | ---------- | ------ |
| **LN dx** | 0.89 | 0.56-1.43 | 0.64 | ------ | ---------- |  |
| **ANA** | **0.24** | **0.071-0.82** | **0.023** | 0.43 | 0.11-1.65 | 0.22 |
| **DNA** | 0.65 | 0.38-1.12 | 0.12 | ------ | ---------- | ------ |
| **Anti-ENA** | 0.94 | 0.52-1.68 | 0.83 | ------ | ---------- | ------ |
| **U1RNP** | **0.62** | **0.38-1.028** | **0.064** | 0.74 | 0.40-1.37 | 0.34 |
| **SSA** | 0.87 | 0.53-1.41 | 0.56 | ------ | ---------- | ------ |
| **SSB** | 0.63 | 0.33-1.17 | 0.14 | ------ | ---------- | ------ |
| **Anti-CCP (binary)**  **≥19 units** | 1.40 | 0.48-4.07 | 0.53 | ------ | ---------- | ------ |
| **CRP (binary)**  **≥10.0 mg/L** | 1.58 | 0.86-2.91 | 0.14 | ------ | ------ | ------ |
| **Current HCQ use** | 0.93 | 0.38-2.26 | 0.87 | 2.15 | 0.71-6.58 | 0.18 |
| **MMF** | 0.84 | 0.49-1.42 | 0.51 | ------ | ---------- | ------ |
| **AZA** | 0.94 | 0.55-1.62 | 0.83 | ------ | ---------- | ------ |
| **Current DMARDs use** | 1.07 | 0.52-2.22 | 0.85 | ------ | ---------- | ------ |
| **Current prednisone use** | **0.49** | **0.21-1.13** | **0.096** | **0.43** | **0.16-1.17** | **0.099** |
| **Cyclophosphamide** | 0.88 | 0.49-1.58 | 0.67 | ------ | ---------- | ------ |
| **Current biologic use** | 0.65 | 0.34-1.24 | 0.19 | ------ | ------ | ------ |
| **Smith** | 0.71 | 0.43-1.18 | 0.19 | ------ | ---------- | ------ |
| **LAC** | 1.03 | 0.56-1.88 | 0.93 | ------ | ---------- | ------ |
| **APS** | 0.78 | 0.33-1.86 | 0.58 | ------ | ---------- | ------ |
| **aCL IgG** | 0.71 | 0.43-1.22 | 0.21 | ------ | ---------- | ------ |
| **aCL IgM** | 0.99 | 0.60-1.67 | 0.99 | ------ | ---------- | ------ |
| **Low c3/c4** | **0.62** | **0.36-1.07** | **0.088** | 0.73 | 0.37-1.43 | 0.36 |
| **Hypertension** | **1.57** | **1.02-2.42** | **0.041** | 1.06 | 0.58-1.92 | 0.86 |
| **Current smoking** | 1.52 | 0.62-3.72 | 0.35 | ------ | ---------- | ------ |
| **Diabetes Mellitus** | **2.87** | **1.35-6.12** | **0.006** | **3.26** | **1.10-9.67** | **0.033** |
| **Current statin use** | **1.77** | **1.03-3.05** | **0.038** | 1.64 | 0.71-3.77 | 0.24 |
| **Current aspirin use** | 0.73 | 0.42-1.26 | 0.26 | ------ | ---------- | ------ |
| **Any QTc meds** | **1.90** | **1.23-2.94** | **0.004** | **1.98** | **1.09-3.59** | **0.024** |
| **Antidepressants** | 1.52 | 0.82-2.85 | 0.19 | ------ | ------ | ------ |
| **Antipsychotics** | 1.80 | 0.64-5.06 | 0.27 | ------ | ---------- | ------ |
| **Antiarrhythmics** | 3.55 | 0.31-40.02 | 0.30 | ------ | ---------- | ------ |
| **Musclerelaxants** | 0.56 | 0.11-2.88 | 0.49 | ------ | ---------- | ------ |
| **Antimicrobials** | 0.99 | 0.54-1.83 | 0.99 | ------ | ---------- | ------ |
| **Antiemetics** | 1.04 | 0.54-1.99 | 0.91 | ------ | ---------- | ------ |
| **Anticonvulsants** | 2.10 | 0.76-5.76 | 0.15 | ------ | ------ | ------ |
| **Tacrolimus Use** | 1.63 | 0.72-3.69 | 0.24 | ------ | ------ | ------ |
| **Ejection fraction** | 0.98 | 0.96-1.01 | 0.30 | ------ | ---------- | ------ |
| **Ejection fraction≥55%** | 0.63 | 0.31-1.25 | 0.18 | ------ | ------ | ------ |
| **Prob>F** | ---------- | | | ***0.025*** | | |

**Abbreviations: *aCL IgG*, Anti-Cardiolipin IgG; *aCL IgM*, Anti-Cardiolipin IgM;  *ANA*, Anti-Nuclear Antigen; *Anti-ENA*, Anti-Extra Nuclear Antigen; *APS*, Antiphospholipid Syndrome; *AZA*, Azathioprine; *Anti-CCP*, Cyclic Citrullinated Peptide Antibody; *CRP*, C-Reactive Protein; *DNA*, Double Strand DNA Antibody; *DMARDs*, Disease-Modifying Anti-rheumatic Drug; *HCQ*, Hydroxychloroquine; *LAC*, Lupus Anticoagulant; *LN dx*, Lupus Nephritis Diagnosis; *MMF*, Mycophenolate Mofetil; *QTcMeds*, QTc prolonging medications; *SSA*, Anti-Sjögren's Syndrome Type A Antibody; *SSB*, Anti-Sjögren's Syndrome Type B Antibody; *Sm*, Anti-Smith Antibody; *U1RNP*, U1 Small Nuclear Ribonucleoprotein Antibody**

**^e^represents final imputed data**

**Table 5. Associations of Clinical Characteristics with QTc≥500 ms in SLE Cohort**

| **Clinical Covariates** | **Univariable Model QTc≥500 ms**  ***OR* 95% CI p** | | |
| --- | --- | --- | --- |
| **Age** | 0.97 | 0.93-1.01 | 0.19 |
| **Sex** | 1.03 | 0.13-8.32 | 0.97 |
| **Race (white vs non-white)** | ---------- | ---------- | ---------- |
| **Disease duration years (square root)** | 0.83 | 0.53-1.31 | 0.43 |
| **LN dx** | 1.59 | 0.47-5.32 | 0.45 |
| **ANA** | ---------- | ---------- | ---------- |
| **DNA** | ---------- | ---------- | ---------- |
| **Anti-ENA** | ---------- | ---------- | ---------- |
| **U1RNP** | 0.56 | 0.13-2.38 | 0.43 |
| **SSA** | 0.63 | 0.14-2.89 | 0.56 |
| **SSB** | 1.33 | 0.25-7.05 | 0.73 |
| **Anti-CCP (binary)**  **≥19 units** | ---------- | ---------- | ---------- |
| **CRP (binary)**  **≥10.0 mg/L** | ---------- | ---------- | ---------- |
| **Current HCQ use** | ---- | ---------- | ---------- |
| **MMF** | 1.064 | 0.26-4.35 | 0.93 |
| **AZA** | 0.55 | 0.11-2.77 | 0.47 |
| **Current DMARDs use** | 1.60 | 0.31-8.26 | 0.57 |
| **Current prednisone use** | 0.60 | 0.072-5.05 | 0.64 |
| **Cyclophosphamide** | 0.31 | 0.037-2.55 | 0.27 |
| **Current biologic use** | 0.39 | 0.047-3.27 | 0.39 |
| **Smith** | **8.89** | **1.06-74.87** | **0.044** |
| **LAC** | ---------- | ---------- | ---------- |
| **APS** | ---------- | ---------- | ---------- |
| **aCL IgG** | 1.51 | 0.33-6.88 | 0.60 |
| **aCL IgM** | 0.74 | 0.14-3.87 | 0.72 |
| **Low c3/c4** | 0.65 | 0.16-2.67 | 0.55 |
| **Hypertension** | 1.77 | 0.51-6.15 | 0.37 |
| **Current Smoking** | 1.71 | 0.21-14.03 | 0.62 |
| **Diabetes Mellitus** | ---------- | ---------- | ---------- |
| **Current statin use** | 1.23 | 0.32-4.70 | 0.76 |
| **Current aspirin use** | 0.67 | 0.17-2.56 | 0.56 |
| **Any QTc meds** | 1.88 | 0.54-6.54 | 0.32 |
| **Antidepressants** | 0.66 | 0.13-3.37 | 0.62 |
| **Antipsychotics** | ---------- | ---------- | ---------- |
| **Antiarrhythmics** | ---------- | ---------- | ---------- |
| **Musclerelaxants** | ---------- | ---------- | ---------- |
| **Antimicrobials** | 0.14 | **0.017-1.21** | **0.075** |
| **Antiemetics** | ---------- | ---------- | ---------- |
| **Anticonvulsants** | ---------- | ---------- | ---------- |
| **Tacrolimus Use** | 4.29 | **0.90-20.33** | **0.067** |
| **Ejection fraction** | 1.12 | 0.91-1.39 | 0.28 |

**Abbreviations: *aCL IgG*, Anti-Cardiolipin IgG; *aCL IgM*, Anti-Cardiolipin IgM; *ANA*, Anti-Nuclear Antigen; *Anti-ENA*, Anti-Extra Nuclear Antigen; *APS*, Antiphospholipid Syndrome; *AZA*, Azathioprine; *Anti-CCP*, Cyclic Citrullinated Peptide Antibody; *CRP*, C-Reactive Protein; *DNA*, Double Strand DNA Antibody; *DMARDs*, Disease-Modifying Anti-rheumatic Drug; *HCQ*, Hydroxychloroquine; *LAC*, Lupus Anticoagulant; *LN dx*, Lupus Nephritis Diagnosis; *MMF*, Mycophenolate Mofetil; *QTcMeds*, QTc prolonging medications; *SSA*, Anti-Sjögren's Syndrome Type A Antibody; *SSB*, Anti-Sjögren's Syndrome Type B Antibody; *Sm*, Anti-Smith Antibody; *U1RNP*, U1 Small Nuclear Ribonucleoprotein Antibody**

**Table 6. Associations of Clinical Characteristics with QTc Length in RA Cohort**

| **Clinical Covariates** | **Univariable Model QTc (Continuous)**  ***Β p*** | | **Multivariable Model QTc (Continuous)**  ***Β p*** | |
| --- | --- | --- | --- | --- |
| **Age** | 0.35 | **<0.10*** | -0.083 | 0.78 |
| **Sex** | 5.19 | 0.23 | -3.87 | 0.64 |
| **Race (white vs non-white)** | 5.24 | 0.203 | 7.07 | 0.27 |
| **Disease duration years (square root)** | -0.010 | 0.99 | ---------- | ---------- |
| **Anti-CCP (binary)**  **≥60 units** | -16.72 | 0.16 | -14.35 | 0.27 |
| **CRP (log)**  **mg/L** | 2.03 | 0.19 | 0.53 | 0.84 |
| **Current use of HCQ** | 7.08 | 0.22 | 18.41 | ***0.047*** |
| **Current use of prednisone** | -3.53 | 0.40 | -4.03 | 0.54 |
| **Current use of biologics** | 0.11 | 0.98 | ---------- | ---------- |
| **Hypertension** | 6.61 | 0.10 | 9.33 | 0.17 |
| **Current smoking** | 8.14 | 0.23 | 8.63 | 0.38 |
| **Diabetes Mellitus** | 3.70 | 0.61 | ---------- | ---------- |
| **Current use of statin** | 2.10 | 0.70 | ---------- | ---------- |
| **Current use of aspirin** | -4.13 | 0.39 | ---------- | ---------- |
| **Any QTc meds** | -1.30 | 0.78 | ---------- | ---------- |
| **Antidepressants** | -3.48 | 0.50 | ---------- | ---------- |
| **Antipsychotics** | -38.27 | 0.10 | -39.58 | 0.10 |
| **Antiarrhythmics** | -8.27 | 0.51 | ---------- | ---------- |
| **MuscleRelaxants** | 3.92 | 0.68 | ---------- | ---------- |
| **Antimicrobials** | 10.40 | 0.53 | ---------- | ---------- |
| **Antiemetics** | -6.13 | 0.65 | ---------- | ---------- |
| **Anticonvulsants** | ---------- | ---------- | ---------- | ---------- |
| **Ejection fraction** | 0.48 | 0.42 | ---------- | ---------- |
| **Ejection fraction>55%** | -10.74 | 0.43 | ---------- | ---------- |
| **Prob>F** | ---------- | | 0.15 | |
| **R-Squared** | ---------- | | 0.12 | |
| **Adjusted R-Squared** | ---------- | | 0.038 | |

**Abbreviations: *HCQ*, Hydroxychloroquine; *QTc meds*, QTc prolonging medications**

**Table 7. Associations of Clinical Characteristics with QTc≥440 ms in RA Cohort**

| **Clinical Covariates** | **Univariable Model QTc≥440 ms**  ***OR 95% CI p*** | | | **Multvariable Model QTc≥440 ms**  ***OR 95% CI* p** | | |
| --- | --- | --- | --- | --- | --- | --- |
| **Age** | 1.00 | 0.98-1.023 | 0.90 | ---------- | ---------- | ---------- |
| **Sex** | **1.99** | **1.18-3.35** | **0.010** | 1.68 | 0.73-3.85 | 0.22 |
| **Race (white vs non-white)** | 1.27 | 0.79-2.05 | 0.32 | ---------- | ---------- | ---------- |
| **Disease duration years (square root)** | 0.99 | 0.85-1.15 | 0.87 | ---------- | ---------- | ---------- |
| **Anti-CCP (binary)**  **≥60 units** | 1.49 | 0.41-5.39 | 0.54 |  |  |  |
| **CRP (log)**  **mg/L** | 1.20 | 1.00-1.45 | ***0.047*** |  |  |  |
| **Current use of HCQ** | 1.09 | **0.54-2.21** | 0.80 | 1.11 | 0.51-2.3  9 | 0.79 |
| **Current use of prednisone** | 0.79 | 0.49-1.29 | 0.35 | ---------- | ---------- | ---------- |
| **Current use of biologics** | 0.72 | 0.45-1.15 | 0.17 | 0.85 | 0.44-1.62 | 0.62 |
| **Hypertension** | 1.05 | 0.65-1.69 | 0.84 | ---------- | ---------- | ---------- |
| **Current smoking** | 1.14 | 0.52-2.47 | 0.75 | ---------- | ---------- | ---------- |
| **Diabetes Mellitus** | 0.83 | 0.38-1.85 | 0.66 | ---------- | ---------- | ---------- |
| **Current use of statin** | 1.029 | 0.54-1.94 | 0.93 | ---------- | ---------- | ---------- |
| **Current use of aspirin** | 0.68 | 0.39-1.20 | 0.18 | 0.95 | 0.37-2.47 | 0.93 |
| **Any QTc meds** | 0.87 | 0.51-1.49 | 0.62 | ---------- | ---------- | ---------- |
| **Antidepressants** | 0.77 | 0.44-1.37 | 0.38 | ---------- | ---------- | ---------- |
| **Antipsychotics** | 0.37 | 0.034-4.19 | 0.43 | ---------- | ---------- | ---------- |
| **Antiarrhythmics** | 0.44 | 0.10-1.90 | 0.27 | ---------- | ---------- | ---------- |
| **MuscleRelaxants** | 1.01 | 0.34-2.99 | 0.98 | ---------- | ---------- | ---------- |
| **Antimicrobials** | 4.68 | 0.56-39.34 | 0.16 | ---------- | ---------- | ---------- |
| **Antiemetics** | 0.37 | 0.067-2.063 | 0.26 | ---------- | ---------- | ---------- |
| **Anticonvulsants** | ---------- | ---------- | ---------- | ---------- | ---------- | ---------- |
| **Ejection fraction** | 0.99 | 0.93-1.06 | 0.88 | ---------- | ---------- | ---------- |
| **Ejection fraction>55%** | 0.43 | 0.086-2.17 | 0.31 | ---------- | ---------- | ---------- |
| **Prob>Chi2** | ---------- | | | 0.73 | | |
| **Pseudo R-Squared** | ---------- | | | 0.013 | | |

**Abbreviations: *HCQ*, Hydroxychloroquine; *QTc meds*, QTc prolonging medications**

**Table 8. Associations of Clinical Characteristics with QTc≥500 ms in RA Cohort**

| **Clinical Covariates** | **Univariable Model QTc≥500 ms**  ***OR 95% CI p*** | | | **Multivariable Model QTc≥500 ms**  ***OR 95% CI p*** | | |
| --- | --- | --- | --- | --- | --- | --- |
| **Age** | **0.95** | **0.92-0.98** | **0.002** | **0.93** | **0.88-0.98** | **0.003** |
| **Sex** | 1.73 | 0.68-4.36 | 0.25 | 0.34 | 0.084-1.39 | 0.13 |
| **Race (white vs non-white)** | 0.59 | 0.27-1.25 | 0.17 | 0.98 | 0.33-2.93 | 0.97 |
| **Disease duration years (square root)** | 0.95 | 0.74-1.22 | 0.69 | ---------- | ---------- | ---------- |
| **Anti-CCP (binary)**  **≥60 units** | 0.26 | 0.066-0.99 | ***0.048*** | 0.16 | 0.033-0.78 | **0.023** |
| **CRP (log)**  **mg/L** | 1.37 | 1.02-1.83 | ***0.035*** | 1.42 | 0.89-2.26 | 0.14 |
| **Current use of HCQ** | 0.54 | 0.18-1.68 | 0.29 | 1.86 | 0.42-8.19 | 0.41 |
| **Current use of prednisone** | 0.91 | 0.41-2.03 | 0.83 | ---------- | ---------- | -------- |
| **Current use of biologics** | 0.91 | 0.43-1.93 | 0.80 | ---------- | ---------- | -------- |
| **Hypertension** | 1.411 | 0.67-2.98 | 0.37 | ---------- | ---------- | ---------- |
| **Current smoking** | **2.62** | **0.97-7.06** | **0.057** | **1.96** | **0.48-8.03** | **0.35** |
| **Diabetes Mellitus** | **4.57** | **1.80-11.64** | **0.001** | **5.86** | **1.31-26.1** | **0.020** |
| **Current use of statin** | 0.55 | 0.16-1.89 | 0.34 | ---------- | ---------- | -------- |
| **Current use of aspirin** | 0.55 | 0.18-1.64 | 0.28 | ---------- | ---------- | -------- |
| **Any QTc meds** | 1.51 | 0.71-3.19 | 0.28 | ---------- | ---------- | ---------- |
| **Antidepressants** | 1.57 | 0.72-3.44 | 0.26 | ---------- | ---------- | ---------- |
| **Antipsychotics** | 3.49 | 0.31-39.38 | 0.31 | ---------- | ---------- | ---------- |
| **Antiarrhythmics** | 0.98 | 0.12-8.16 | 0.98 | ---------- | ---------- | ---------- |
| **MuscleRelaxants** | 1.15 | 0.25-5.34 | 0.86 | ---------- | ---------- | ---------- |
| **Antimicrobials** | **5.48** | **1.18-25.48** | **0.030** | 1.29 | 0.054-30.99 | 0.87 |
| **Antiemetics** | ---------- | ---------- | ---------- | ---------- | ---------- | ---------- |
| **Anticonvulsants** | ---------- | ---------- | ---------- | ---------- | ---------- | ---------- |
| **Ejection fraction** | 0.99 | 0.90-1.08 | 0.78 | ---------- | ---------- | ---------- |
| **Ejection fraction>55%** | 0.42 | 0.083-2.18 | 0.31 | ------- | ---------- | -------- |
| **Prob>Chi2** | ---------- | | | ***0.015*** | | |
| **Pseudo R-Squared** | ---------- | | | 0.17 | | |

**Abbreviations: *HCQ*, Hydroxychloroquine; *QTc meds*, QTc prolonging medications**

**Table 9. Interactions in RA Cohort**

| **QTc** | ***Β*** | **p** |
| --- | --- | --- |
| **Current HCQ Use#AnyQTcMeds** | -5.53 | 0.68 |
| **Current HCQ Use#Antidepressants** | -15.9 | 0.26 |
| **Current HCQ Use#Antipsychotics** | -36.3 | 0.26 |
| **Current HCQ Use#Musclerelaxants** | 1.10 | 0.97 |
| **Current HCQ Use#Antimicrobials** | 6.17 | 0.88 |
| **Current HCQ Use#Antiemetics** | -53.4 | 0.15 |

**Table 10. Interactions in SLE Cohort**

| **QTc** | ***Β*** | **p** |
| --- | --- | --- |
| **Current HCQ Use#AnyQTcMeds** | -0.33 | 0.98 |
| **Current HCQ Use#Antidepressants** | 13.6 | 0.29 |
| **Current HCQ Use#Antipsychotics** | 0.86 | 0.93 |
| **Current HCQ Use#Antimicrobials** | 2.31 | 0.85 |
| **Current HCQ Use#Antiemetics** | 6.06 | 0.64 |
| **Current HCQ Use#Tacrolimus** | 22.2 | 0.39 |
